# Parvovirus 4 in Blood Donors, France

**DOI:** 10.3201/eid1601.090517

**Published:** 2010-01

**Authors:** Mhammed Touinssi, Nadège Brisbarre, Christophe Picard, Coralie Frassati, Bertrand Dussol, Rathviro Uch, Pierre Gallian, Jean-François Cantaloube, Philippe de Micco, Philippe Biagini

**Affiliations:** Université de la Méditerranée, Marseille, France (M. Touinssi, N. Brisbarre, R. Uch, P. Gallian, J.-F. Cantaloube, P. de Micco, P. Biagini); Service Immunogénétique-HLA, Etablissement Français du Sang Alpes-Méditerranée, Marseille (C. Picard, C. Frassati); and Centre de Néphrologie et de Transplantation Rénale, CHU, Marseille (B. Dussol)

**Keywords:** PARV4, Parvovirus 4, blood donors, viruses, France, letter

**To the Editor:** In the past few years, several novel parvoviruses have been identified, including human parvovirus B19–related strains V9 and A6, and bocavirus. In 2005, parvovirus 4 (PARV4), a new putative member of the family *Parvoviridae*, was identified in the plasma of a patient in North America who had an acute virus infection (*1*). This virus had limited sequence homology with parvovirus B19 (<30% aa similarity) despite a conserved genomic organization showing 2 large nonoverlapping open reading frames (ORF). Phylogenetic studies performed with near-complete sequences have proposed that human PARV4 can be described by ≥3 genogroups (*2*).

Little information is available about the natural history of the virus, and its clinical role remains unknown. Initial studies showed that PARV4 was present in the blood of febrile patients, intravenous drug users, and persons positive for hepatitis C virus or HIV at prevalences of 6%–30% (*3**–**5*). Two recent studies also showed the virus in cohorts of kidney transplant patients (*6**,**7*). PARV4 has also been identified in persons without apparent pathologic changes, such as blood donors (1.0%–3.95%), and in blood products negative for parvovirus B19 DNA (*4**–**6*). Virus DNA also was found in bone marrow and various tissues or organs, suggesting possible dispersion of the virus in diverse biologic locations (*8*).

To provide new insights into the dispersion of PARV4 in healthy persons, we assessed the frequency of PARV4 viremia in a cohort of blood donors by using a dual real-time assay. A total of 304 volunteer blood donors (154 men; mean age of all volunteers 40 years) living in southeastern France entered the study. The sex ratio (men:women: 1.03) and age distribution (19–65 years) of the cohort were considered representative of the population of blood donors at that time. Blood samples were collected in vacuum tubes (Vacutainer, SST, Becton Dickinson, Meylan, France) and centrifuged, and 1-mL plasma aliquots were stored at −80°C until use. Nucleic acids were extracted as described previously (*7*) and tested for PARV4 genomic DNA immediately after extraction to avoid freezing/thawing cycles.

Five microliters of the eluted material served for PARV4 DNA detection using real-time PCR TaqMan methods (StepOne Plus, Applied Biosystems, Courtaboeuf, France) and primers (ORF2) compatible with the detection of the 3 virus genogroups described at this time (*2**,**4**,**7*). Two probes were tested in separate amplification assays: PARV4-O (5′-FAM-TGTTCAACTTTCTCAGGTCCTACCGCCC-TAMRA-3′) (*4**,**7*) and PARV4-N (5′-FAM-TCCTACYGCCCSCTCCTCCTTCTT-TAMRA-3′). The second primer was designed after identification and sequencing of several in-house real-time PCR products (GenBank accession nos. FJ883557–61), and more particularly those detectable on agarose gels but showing negative signal with PARV4-O TaqMan assay because of mismatches identified on the probe recognition site (Figure). Amplification reactions were performed as described previously (*7*). Both TaqMan assays were estimated to detect as few as 10 copies of PARV4 DNA per reaction using dilutions of a synthetic template (*7*).

**Figure:**
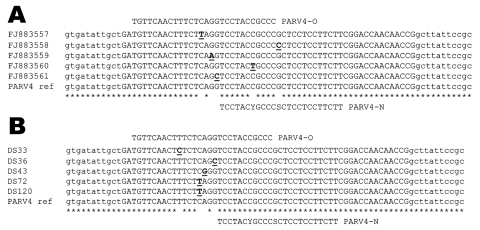
Alignment of parvovirus 4 (PARV4) sequences showing the location of the 2 probes used in the real-time experiments. A) Partial sequences used for the design of probe PARV4-N: PARV4 prototype isolate (AY622943) and in-house PCR products characterized initially (GenBank accession nos. FJ883557–61). B) Examples of point mutations located on the PARV4-O recognition site identified in amplicons originating from samples positive with PARV4-N assay. Mismatches identified in the alignments are underlined. Nucleotides shown in lowercase letters correspond to 5′/3′ ends of the real-time primers. Mismatches identified in the alignments are underlined and in **boldface**.

Fifteen (4.9%) blood donors gave positive signal with probe PARV4-O; 62 (20.4%) were positive with probe PARV4-N; 6 (2.0%) samples were positive in both assays. Overall prevalence for PARV4 DNA was 24.0% in the blood donors tested.

PARV4 origin of randomly selected PCR products from PARV4-O and PARV4-N TaqMan assays (5 each) was confirmed after cloning and M13 sequencing. Point mutations located on the PARV4-O recognition site were retrieved in amplicons originating from samples positive with PARV4-N assay (Figure). However, subsequent molecular studies aiming to characterize virus strains were not feasible because of the low titer of PARV4 DNA (<500 copies/mL of plasma) in positive samples. No specific correlation was identified between sex or geographic origin and PARV4 viremia, whereas analysis of the distribution of PARV4-positive samples in age groups highlighted a relative homogeneity throughout the corresponding cohort.

Our study shows that PARV4 infection is readily detectable in French blood donors. Prevalence results using probe PARV4-O were comparable to those obtained in previous studies involving healthy persons originating from various countries (*4**–**6*). Conversely, the high prevalence obtained by using probe PARV4-N was unexpected because only 1 study demonstrated a higher value (45.7%) after the investigation of PARV4 DNA in bone marrow aspirates of AIDS patients from Italy (*9*).

This finding suggests a larger dispersion of PARV4 than expected initially in the general population and highlights the need for improvement in detection systems directed toward PARV4 DNA, particularly by interlaboratory collaborations, in direct connection with studies investigating PARV4 genetic diversity. These considerations are consistent with the recent description of a new PARV4 genogroup in humans and characterization of highly divergent variants in bovine and porcine species (*10*). In addition, such data raise the question of the consequent persistence of PARV4 infection in healthy persons. Future studies need to explore both dispersion and potential clinical impact of PARV4 on infected hosts.
